# The Effect of Labelling and Visual Properties on the Acceptance of Foods Containing Insects

**DOI:** 10.3390/nu12092498

**Published:** 2020-08-19

**Authors:** Klaudia Modlinska, Dominika Adamczyk, Katarzyna Goncikowska, Dominika Maison, Wojciech Pisula

**Affiliations:** 1 Institute of Psychology, Polish Academy of Sciences, 1 Jaracza St., 00-378 Warsaw, Poland; wojciech.pisula@wp.pl; 2 Faculty of Psychology, University of Warsaw, 5/6 Stawki St., 00-183 Warsaw, Poland; dominika.adamczyk@psych.uw.edu.pl (D.A.); katarzyna.goncikowska@gmail.com (K.G.); dominika@psych.uw.edu.pl (D.M.)

**Keywords:** food labelling, entomophagy, insect-based foods, edible insects, food sustainability, perception of food, novel food, disgust, neophobia, variety seeking, food technology neophobia, consumer studies, behavior

## Abstract

Introducing insects as a source of nutrients (e.g., protein) plays a key role in many countries’ environmental policies. However, westerners generally reject insects as an ingredient of food products and meals. The aim of our study was to assess if explicitly labelling food as containing insects and/or implying it by manipulating the appearance of food influences the participants’ perception of food products or their behavioral reaction to such products. Participants were asked to try a range of foods, none of which contained ingredients derived from insects. However, the experimental conditions varied with regard to food labelling (insect content) and appearance (traces of insect-like ingredients). We observed the participants’ non-verbal behavioral reactions to the foods. Next, the respondents filled in a questionnaire evaluating the food’s properties. Additionally, we asked the participants to fill in a set of questionnaires measuring other variables (food neophobia, disgust, variety seeking, etc.) The results showed that products labelled as containing insects are consumed with reluctance and in lower quantities despite their appearance. In addition, people with lower general neophobia and a higher tendency to seek variety tried the insect-labelled samples sooner than people from the other groups. Recommendations for marketing strategies are provided.

## 1. Introduction

While food shortages mainly affect developing countries, where malnutrition is a problem for millions [1], in highly developed countries the conventional methods of food production burden the environment and pose a threat to animal welfare. Introducing insect-based food products could contribute to solving both issues [2].

Entomophagy is present in many cultures and is the main source of nutritious food for many communities [3]. Insects are a source of protein (and amino acids), fats, vitamins (e.g., vitamin B12), beta-carotene, several minerals, fiber and other valuable substances [4,5,6,7,8]. Insect production requires less space [9] and leaves a significantly smaller ecological footprint than livestock farming, and is more ecologically sustainable [10,11]. Moreover, as insects are evolutionarily distant from humans, they are less likely to carry pathogens that could pose a risk to human health [7,12].

Although nowadays the consumption of insects by humans is regularly discussed in the media, attempts at changing people’s eating habits and introducing these new types of food have so far generally met with individual and social rejection. Although some recent studies show a positive effect of information on the willingness to try insect-based foods (e.g., [13,14,15,16]), it seems that, in general, rational arguments stressing the advantages of insect production and consumption are not sufficient to change our eating habits [17,18] (see also [19]). In general, westerners are largely opposed to eating insects, as they are an unfamiliar food source that deviates from cultural norms and are expected to have undesirable sensory properties [20] (cf. [21]).

It seems that the main psychological barriers to consuming this kind of novel food are disgust and fear [22,23,24]. Most people object not only to eating insects, but also to the very idea of eating insects [22,23], and their negative reactions to insects may be very deeply rooted and automatic [25]. The first explanation for this reaction is disgust. Disgust is an emotion that elicits thoughts and behaviors that result in avoiding the objects that trigger it [26]. Things that elicit disgust differ largely from one culture to another; they are acquired in the learning process and are deeply rooted in social norms. Disgust correlates with the evolutionary need to avoid infection, dirt and disease, and may lead to fear [23]. In the case of food, this fear is manifested in anxious hesitation to consume unknown items, which is called food neophobia [27]. Food neophobia correlates positively with general neophobia [27], which may increase reluctance towards unfamiliarity. Therefore, it is likely that persons with a high level of food neophobia, as well as a high level of general neophobia, would be prone to the feeling of disgust (see [28,29,30]). Persons who fit this profile are very likely to be the least willing to include insects in their diet [18]. Moreover, in the case of such new commercially available products as insects and insect flour, and their mostly unknown production processes, Food Technology Neophobia [31] may also play a crucial part. On the other hand, it is possible that other characteristics such as the Variety Seeking Tendency [32], which involves an intrinsic desire for variety in many aspects of life, including food consumption, may increase the willingness to try new food products [33].

Although the main psychological variables influencing the acceptance/rejection of insect-based foods seem to have been identified, the problem of a limited willingness to incorporate such foods into everyday diet has not been resolved. Over the past few decades, researchers have been trying to devise a strategy to convince people that insects are indeed edible, safe, and tasty. It may be hypothesized that one such strategy could involve adapting foods containing insects to the consumers’ general eating preferences. It is clear, for instance, that consumers prefer fatty and sweet products (e.g., [34,35]). Serving insects in the form of, or as an ingredient of, snacks or sweet foods may increase their acceptance and create positive associations with such types of food, facilitating habituation to insect-based foods. On the other hand, studies by Tan et al. [36] showed that sweet-flavored insects were considered less appropriate than savory insect-based meals, especially when the entire insect body or insect body parts were easily discernible.

Another way of reducing the level of food neophobia may be to add insects to familiar dishes [14] (cf. [37]). However, the form in which they are added also plays a part. It seems that adding ground insects to a meal could reduce reluctance to consume it by reducing exposure to the visual stimulus (e.g., [20]). In the case of unconventional animal-derived foods, evoking the image of the entire animal often elicits strong objection to the food offered and is connected with strong negative emotions [38]. When eating animals, we usually consume pieces that do not resemble living individuals; we are reluctant to eat animal heads, entire limbs, etc. Perhaps in the case of insects, the fact that they are often served whole elicits similar reactions in humans by making it clear what they are about to consume. Moreover, adding entire insects to meals may give the impression that a food is polluted or rotten. Evidence to support this claim has been provided in a study by Gmuer et al. [39], which showed that potato chips which contained insect flour or insect elements were assessed less negatively than chips mixed with whole insects (see also [29,40,41]). Other studies, however, show no such correlation (e.g., [42,43]). In addition, the widely applied marketing strategy of presenting insects as sustainable substitutes for animal protein is also problematic, as it gives rise to an expectation that the products will be similar to meat in texture and taste, as is the case with plant-based products made to look similar to their meat-based counterparts [17] (cf. [30]).

Nevertheless, it seems that the reluctance to eat insects is so strong that the very awareness of consuming them elicits an aversive reaction, which may then be generalized to other accompanying products. This hypothesis seems to have been confirmed by Rozin et al. [44] in a study where participants assessed a drink more negatively if the cup they drank from had been in contact with a sterile insect before it was used. Such a reaction may be explained by the participants’ feeling that the cup had been contaminated during contact with the insect, and the disgust triggered by that perception was generalized to the drink. In the case of commercially available products, information on insect content is provided in visual form on packaging or it is provided in the product name, which may have a similar effect. Studies carried out to date have shown that both verbal and visual information on packaging has a significant impact on how consumers evaluate the taste and smell of the substances they ingest (e.g., [45,46,47,48]). The very indication of insect content on the packaging may affect consumers’ assessment of the product quality. Moreover, it may be assumed that ingredients whose appearance suggests the presence of traces of insects in a product may additionally reinforce the label effect by reinforcing the impression that the product has been contaminated (cf. [23]).

Based on the above, we are convinced that more research is needed to further explore the factors influencing peoples’ attitude to eating insect-based foods and to identify marketing strategies and educational campaigns. We would like to propose the study protocol involving an assessment of several variables that have been identified in other studies (the results of which, however, are in some cases inconclusive) or that stem from our predictions. In our study, we intended to check, first of all, whether the mere fact of providing information about insect content (label) would influence the sensory evaluation and acceptance of different foods. In this way, we wanted to replicate the findings obtained by Mancini and colleagues [14]. However, we decided to use sweet foods (pastry, sweets), as we hypothesized that snacks would be more likely to encourage people to try this kind of food, especially bearing in mind the general preference for sweetness among consumers. Secondly, we hoped to broaden the scope of the study by evaluating the effect of visibility of insect parts on the level of acceptance of insect-based foods, as studies that have analyzed these aspects have been inconclusive. We expected that the differences in the results obtained by other researchers could have arisen from a specific “presentation” of insects, but also from an interactive effect of the information on insect content and the appearance of food. In addition, we planned to control the mediating effect of commonly studied psychological variables, such as food neophobia and disgust. However, we also incorporated other measures in the study; we used the food technology neophobia scale and the variety-seeking tendency scale to further explore the mechanism underlying the acceptance of insect-based food.

We intended to analyze these aspects by checking experimentally reactions to food products depending on (a) the information about insect content provided on product labelling, and (b) the appearance of products suggestive of traces of insect-like ingredients. To address these issues, we conducted an experiment whereby we observed participants’ reactions to specific products: a cookie, a muffin, and a date ball. The experiment resembled a classic product test in which the consumers tried and assessed three products in terms of taste, smell, appearance, etc. Depending on the experimental conditions (2 × 2), the products differed either (a) with regard to the information about the presence or absence of insect content, or (b) in appearance: they are artificially “dotted” to suggest insect content or not. In reality, none of the products used in the experiment contained insects or insect-derived ingredients.

Another important novel element of our experiment, when compared to many previous studies, was the fact that we not only measured participant reactions in qualitative scale-based terms (declaration of willingness to taste a specific product), but we also observed several non-verbal behavioral indicators (e.g., we measured the time and frequency of sampling products and how much of the product was ingested). What is more, after the experiment, we conducted interviews with the participants, which served as a source of additional information to enrich the discussion section of the paper and allow us to suggest possible marketing strategies.

Based on the results of previous studies, we formulated the following hypotheses:

**Hypothesis** **1 (H1).** *Information about insect-based ingredients on the product label will translate into lower product evaluation scores and will have an impact on the behavioral reactions to the product (cf. [44,45,46,47,48]).*

**Hypothesis** **2 (H2).** *Product appearance (traces of ingredients suggesting insect content) will translate into lower product evaluation scores and will have an impact on the behavioral reactions to a product (e.g., [20,29,39,41]).*

**Hypothesis** **3 (H3).** *The perception of the product and behavioral indicators will be controlled by different psychological features. People with higher food neophobia, sensitivity to disgust, food technology neophobia, and general neophobia are expected to be less willing to try products described as containing insect-derived ingredients (product evaluation and behavioral reactions to the products), while people with a high level of variety-seeking tendency will be more open to this kind of novel food (e.g., [22,23,32,43]).*

## 2. Methods

### 2.1. Participants

The participants were recruited by means of announcements posted online, on website panels, and individual personal requests. All participants gave their oral consent to participate in the study. They also confirmed that they did not suffer from any form of food allergy or intolerance. One person was excluded from the study after having a strong emotional reaction to the food samples and after disclosing an existing psychiatric condition. The final sample comprised 99 participants (81 females and 18 males). The participants were aged between 18 and 45 years old, while the average age was 22. They were mostly university students who came from big cities (>50,000 inhabitants).

The participants were randomly assigned to four experimental groups. In each group, the male to female ratio was comparable. They were told that the purpose of the experiment was to study food preferences. Before the experiment, they were not informed that the experiment involved tasting insect-based products, so their attitudes and expectations did not affect the answers given in the questionnaires and the participants who tasted products with no indication of insects did not feel they were being misled, or that the products they were supposed to try contained hidden insect parts. The data was collected anonymously. Each person was assigned an individual number to allow the data from all parts of the experiment to be linked.

### 2.2. Procedure and Methods

The study consisted of three parts. In Part 1 the participants were asked to fill in a set of questionnaires. In Part 2 a behavioral experiment was conducted. Part 3 involved conducting a short interview with the participants.

Part 1—Questionnaires

The participants answered a set of questionnaires preceded by socio-demographic questions. They were also asked about their diet preferences (whether they followed a meat or non-meat diet). This information was collected to control the characteristics of the experimental groups and to avoid any possible impact of this variable on the behavioral data collected in Part 2. All questionnaires were administered in a paper-and-pen form. The time to complete the questionnaires was not limited.

Part 2—Behavioral assessment

**Figure 1:**
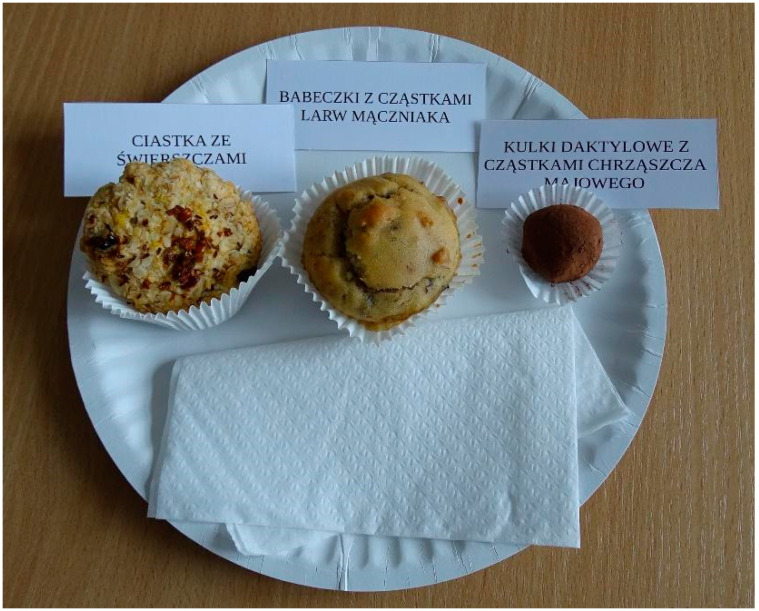
A set of three food products: (from left to right:) a cookie, a muffin, a date ball. Labels attached to food products: **Group 1A:** Rice flour cookie; amaranth flour cupcakes; plain date balls—products labelled as not containing insects. **Group 2A:** Cricket flour cookie; mealworm flour cupcakes; beetle flour date balls—products labelled as containing insects. **Group 1B:** cookies with chunks of cranberries; cupcakes with chunks of walnuts; date balls with linseed—products labelled as not containing insects. **Group 2B:** Cookies with crickets; cupcakes with particles of mealworm larvae; date balls with May beetle particles—products labelled as containing insects.

After completing the questionnaires, the participants were asked to move to another room, where behavioral assessment was conducted. They were seated at a small table and received instructions about the experimental procedure. The participants were randomly assigned to one of four experimental set-ups (Table 1). Each person received a set of three food products (A—a cookie, B—a muffin, C—a date ball). The products were placed on a paper plate, and a label with the name of the product was placed above each item (Figure 1). In two of the four groups, the labels explicitly stated that the products contained insects and in the two other groups, there was no information about insect content. In addition, half of the participants were given samples that could suggest the presence of mildly processed insects in the food (e.g., chunks of cranberries or nuts easily discernible in the products), while the other half were served “smooth” products. The experimental group set-up followed the ANOVA 2 × 2 procedure (Table 1).

The participants were asked to try the products in a given order. The questionnaires assessing the properties of each of the three products were placed above the plate in front of each participant. The questionnaire concerned the respondents’ willingness to try the product; the visual attractiveness of the product; smell; taste; and willingness to eat more of the product. The respondents were asked to indicate their answers on a 10-point linear scale with descriptive explanations provided at the end-points. In addition, each participant received a napkin and a cup filled with water. The participants were told that they were free to eat as much of any of the products as they wanted, and that they were free not to try any of them if they did not want to.

Each participant’s behavior during the food-tasting session was recorded with a video camera placed approx. 2 meters in front of the participant.

Part 3—Interview

After the tasting session, the participants moved to an adjacent room for a short qualitative semi-structured individual interview. The interviews were aimed at broadening the scope of data collected in the study and at providing a context for the quantitative data collected. The scenario comprised a few precisely defined thematic areas, but the questions pertaining to those areas were not pre-determined and they were individually adapted to the respondent and interview dynamics.

The aim of the interview was to examine the opinions and experiences of the respondents with respect to entomophagy; to gain a more in-depth knowledge about the participants’ experience of the food-tasting session; to understand the motivations behind the (un)willingness to try the products; and to examine the declared readiness to include insects in the daily diet and the participants’ view on the social aspects of insect-based diets. The participants were asked about their previous experience with entomophagy (e.g., whether they had ever heard of this phenomenon before, whether they had ever tried insects, whether they believed that insects could be part of a normal human diet and what potential consequences of such a diet could be). Next, the participants were asked about their impressions from the tasting session and the likelihood of including insects in their own diet. The interviewees were encouraged to elaborate on their answers so that a wider range of information could be collected.

The interviews were recorded and transcribed. 

### 2.3. Ethical Statement

All subjects gave their informed consent for inclusion before they participated in the study. The study was conducted in accordance with the Declaration of Helsinki, and the protocol was approved by the Commission of Ethics of Scientific Research of the Faculty of Psychology, University of Warsaw, Poland (No. 21/03/2019). Additionally, the participants signed a consent form agreeing to the processing of their personal data (including audio and video recordings). They were also assured that they could withdraw from the experiment at any point of the procedure and that all the data collected during the study (including the recordings) would only be available to the research team members.

### 2.4. Questionnaires

All the questionnaires were translated from English into Polish using the back-translation method [49,50]. First, the questionnaires were translated from English into Polish by a professional translator, then a different translator translated the Polish version back into English. The original English and the back-translated versions were compared by a native English speaker. The text was edited according to the native English speakers’ comments, following which the reformulated items were translated into English by a translator not familiar with the content of the comments or the original questionnaire; they were then compared with the original by a native English speaker. The final Polish versions of the questionnaires were then consolidated. The internal consistency of the Polish versions of the questionnaires was determined using Cronbach’s alpha.

#### 2.4.1. FNS

The Food Neophobia Scale (FNS), developed by Pilner and Hobden [33], is often used to measure the willingness to try new foods [51]. It consists of ten items on a scale from 1 (“strongly disagree”) to 7 (“strongly agree”); five statements are positive (indicative of neophilic attitudes) and five are negative (indicative of neophobic attitudes). Examples of statements include: “I don’t trust new foods” and “At dinner parties, I will try a new food”. The internal consistency of the Polish version of the scale in our study measured using Cronbach’s alpha was estimated at 0.87.

#### 2.4.2. GNS

The General Neophobia Scale (GNS) is a scale measuring the general level of neophobia. It was developed by Pliner and Hobden [33] together with the Food Neophobia Scale. It is an eight-item 7-point Likert scale from 1 (“strongly disagree”) to 7 (“strongly agree”). Examples of statements include: “I am afraid of the unknown” and “Whenever I am on vacation, I can’t wait to get home”. The internal consistency of the Polish version of the scale in our study measured using Cronbach’s alpha was estimated at 0.91.

#### 2.4.3. FTNS

The Food Technology Neophobia Scale (FTNS; [31]) is a tool suitable for measuring the willingness to try new food products manufactured using new food technologies and attitudes to new food technologies. It consists of thirteen items scaled from 1 (“strongly disagree”) to 7 (“strongly agree”). Examples of statements include: “It can be risky to switch to new food technologies too quickly” and “New products produced using new food technologies can help people have a balanced diet”. The internal consistency of the Polish version of the scale in our study measured using Cronbach’s alpha was estimated at 0.88.

#### 2.4.4. The Disgust Sensitivity Scale

The Disgust Sensitivity Scale (Version 1; [52]) is a measure of individual differences in sensitivity to disgust. It is a scale with thirty-two items: the first sixteen are binary “true” or “false” questions, the rest assess how disgusting a given experience seems on a scale from 0 (“not disgusting at all”) to 2 (“very disgusting”). Example items include: “Even if I was hungry, I would not drink a bowl of my favourite soup if it had been stirred by a used but thoroughly washed flyswatter” and “Seeing a cockroach in someone else’s house doesn’t bother me”. The internal consistency of the Polish version of the scales in our study measured using Cronbach’s alpha was estimated at 0.825.

#### 2.4.5. VARSEEK-Scale

The Variety Seeking Tendency Scale (VARSEEK-Scale; [32]) is a scale measuring individual variety-seeking tendency in food choices [53]. The respondents assessed eight statements on a five-point Likert scale (from “completely disagree” to “completely agree”). Examples of statements include: “I prefer to eat food products I am used to” and “When I eat out, I like to try the most unusual items, even if I am not sure I would like them”. The internal consistency of the Polish version of the scale in our study measured using Cronbach’s alpha was estimated at 0.89. 

### 2.5. Data Processing and Statistical Analysis

In addition to using the questionnaires measuring specific psychological traits, we also analyzed the behavioral data collected during the experimental (product tasting) phase. This data was analyzed on the basis of video recordings made during the experiment. We used BORIS software [54] to code behaviors on the basis of the recorded material, which made it possible to define selected behaviors and to assess their duration and frequency. We scored the behaviors the participants engaged in during the entire experimental phase. Consequently, we were able to assign specific scores to the duration of separate bouts of behavior, their frequency, and the total time participants spent engaging in specific behaviors. We measured the following variables: latency to pick up food products from the plate; latency to begin eating; amount of food eaten (for each product separately); time spent eating; time and frequency of sniffing food products, time and frequency of looking at the food products; frequency of drinking water during the tasting session. In addition, we analyzed the scores awarded by the participants for the products tasted.

The measurements of different behaviors taken in the course of the experiment (as a response to the exposure to food products) were analyzed using an analysis of covariance (ANCOVA) with Label (2) × Appearance (2) as between-subject factors. The behavioral measurements served as dependent variables, while Label and Appearance manipulations stood for independent variables. Scores from the questionnaires served as covariates. For multiple comparisons, the Bonferroni correction was applied to reduce the likelihood of Type I error. Differences were considered significant for *p* values of <0.05. 

For the amount of food eaten, a repeated measures ANOVA was conducted to analyze the effect of possible differences between the three food products (A—cookie, B—muffin, C—date ball) on a given variable. Label (2) × Appearance (2) were used as between-subject factors and Sample as the within-subject factor (3). Scores obtained from the questionnaires served as covariates.

Descriptive statistics are set out in Appendix A.

The audio data collected during the interviews was transcribed and then subjected to the thematic analysis, which is a method for developing qualitative data consisting of identification, analysis and description of thematic areas [55]. In this type of analysis, a thematic unit is treated as an element related to the research problem that includes an important aspect of data. Two interview moderators, i.e., persons responsible for interviewing the respondents, encoded and analyzed the transcriptions. Next, the created codes were cross-checked. An important advantage of thematic analysis is its flexibility, which makes it possible to adopt a research strategy best suited to the phenomenon under examination. In our study, the thematic analysis focused on various aspects of insect consumption and general attitudes towards insects. However, account was taken of the exploratory nature of the study and the novelty of the phenomenon, and thus the low level of the participants’ familiarity with the topic. 

## 3. Results

Characteristics of the participants. First, we checked the characteristics of the study participants, especially whether they were distributed equally between the four groups with respect to the demographic variables and the basic psychological measures included in the study (Table 2). Statistical analysis showed no significant differences between the four experimental set-ups with regard to gender balance, age, level of education, and psychological variables. Even though the number of women was four times higher than the number of men, the ratio of men to women was the same in each set-up.

### 3.1. Effect of Label and Appearance on Food Acceptance (Behavioural Data)

Latency to pick up food. The analysis of the latency to pick up the food products from the plate showed a main effect of Label (F(1, 89) = 6.456, *p* = 0.013, eta^2^ = 0.068). Products labelled as containing insects were picked up later than those with labels not indicating any insect content (t = 2.541, *p* = 0.013, d = 0.507). There was no interactive effect of Label and Appearance, nor a main effect of Appearance. We also found main effects of the covariates General Neophobia (F(1, 89) = 5.825, *p* = 0.018, eta^2^ = 0.061) and Variety Seeking (F(1, 89) = 6.896, *p* = 0.01, eta^2^ = 0.072). There was a positive correlation between the latency to pick up food and General Neophobia scores (r = 0.206, *p* < 0.05), while a negative correlation was observed for Variety Seeking Tendency (r = −0.219, *p* < 0.05). This may suggest that participants with a higher level of general neophobia started eating food samples later than those with a lower level of general neophobia. Participants with a higher level of variety seeking tendency started eating sooner than those demonstrating a lower level of this characteristic.

Latency to begin eating. In the case of latency to begin eating, we observed a main effect of Label (F(1, 88) = 5.570, *p* = 0.020, eta^2^ = 0.059). Participants began to digest food samples labelled as containing insects later than those with labels not indicating any insect content (t = 2.360, *p* = 0.020, d = 0.503). There was no interactive effect of Label and Appearance, nor a main effect of Appearance. There was no effect of covariates.

Sniffing and looking at products. Analyses of the time and frequency of sniffing the food products and the time and frequency of looking at the food products showed no differences between the groups, which means that participants examined olfactory and visual properties of the samples for a comparable amount of time despite their different labels and appearances. No effect of covariates was found.

**Figure 2:**
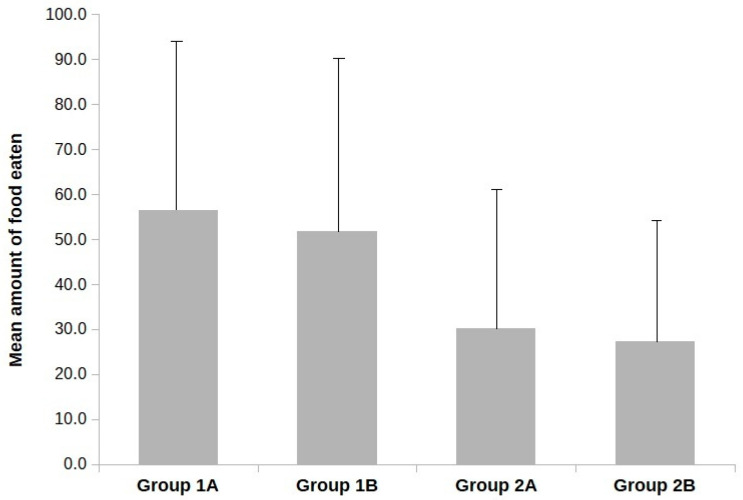
Mean amount of food eaten in each group.

Amount of food eaten. A repeated measures ANOVA for the amount of food eaten revealed a main effect of Label for all three food samples (F(1, 90) = 23.918, *p* = 0.001, eta^2^ = 0.210), but there was no main effect of Appearance or Sample – Figure 2. There were no interactive effects of Sample and Label, nor an interactive effect of Sample and Appearance. This may indicate that the food samples were found to be equally tasty, and the effect of Label was similar for all the samples. Samples labelled as containing insects were consumed in lower quantities then those with labels not indicating insect content regardless of the type of food (sample A: t = 6.282, *p* < 0.001, d = 0.884; sample B: t = 2.637, *p* < 0.001, d = 0.551; sample C: t = 4.646, *p* < 0.001, d = 0.963). A significant effect of the Food Neophobia covariate was also found (F(1, 90) = 4.283, *p* = 0.041, eta^2^ = 0.045). The correlation between Food Neophobia scores and the amount of food eaten was found to be negative, but only for sample C (r = −0.21, *p* = 0.037).

Time spent eating. An ANCOVA analysis conducted for the time spent eating yielded a significant effect for Label (F(1, 89) = 5.922, *p* = 0.017, eta^2^ = 0.062), with participants exposed to meals labelled as containing insects eating for a shorter time than individuals who were offered samples with labels not indicating insect content (t = −2.433, *p* = 0.017, d = 0.527). There was no main effect of Appearance, nor an interactive effect of Label and Appearance.

A similar effect was observed in the case of the frequency of eating bouts. The analysis showed only a main effect of Label (F(1, 89) = 10.541, *p* = 0.002, eta^2^ = 0.106). Participants who ingested food labelled as containing insects ate less frequently than those who were offered food with labels not indicating insect content. These two results may be correlated, as the shorter consumption time probably involves less frequent bites. No effect of covariates was observed.

*Frequency of drinking water.* Differences between the groups were observed in the frequency of drinking water when ingesting food. An ANCOVA revealed an interactive effect of Label and Appearance (F(1, 89) = 4.625, *p* = 0.034, eta^2^ = 0.049). A post-hoc analysis showed that participants who ate the samples with labels and appearance not indicating insect content drank water more frequently than those who ate the products labelled as containing insects with a matching appearance (t = 3.214, *p* = 0.011, d = 0.793) and than those who ate the products labelled as containing insects with a “smooth” appearance (t = 2.906, *p* = 0.028, d = 0.121). No effect of covariates was observed.

The above results support the first hypothesis. The effect of Label was found in the latency to pick up food, latency to begin eating, amount of food eaten, and time spent eating variations. Food labelled as containing insects was tasted later and in smaller quantities than food labelled as not containing insects.

However, we found no support for the second hypothesis. There were no differences in the behavioral measures between the foods with traces of insect-like parts and those with a smooth appearance.

Additionally, no interactive effect of Label and Appearance was observed.

The third hypothesis was only partially confirmed. The level of food neophobia correlated only with the amount of food eaten for sample C. General neophobia level and variety seeking tendency level correlated with the latency to pick up food. However, we did not find any confounding effect of disgust and food technology neophobia.

### 3.2. Influence of Label and Appearance on Food Evaluation (Product Questionnaires)

We conducted an ANCOVA analysis of food evaluation scores depending on product information (label) and food appearance. For the first question about the willingness to try the products offered, there was a main effect of Label (F(1, 89) = 5.379, *p* = 0.023, eta^2^ = 0.056) and a main effect of Appearance (F(1, 89) = 4.460, *p* = 0.037, eta^2^ = 0.047), but no interactive effect was observed. Participants declared less willingness to try the products labelled as containing insects (t = 2.319, *p* = 0.023, d = 0.471) and the “smooth” products with no easily discernible elements (t = 2.112, *p* = 0.037, d = 0.414). However, there were no differences between the experimental groups with regard to the scores awarded for appearance, smell, taste, and willingness to eat more of the product. There was no effect of covariates.

These findings support the first and second hypotheses, but only as regards the first question of the questionnaire.

### 3.3. Qualitative Data Analysis—Interviews

Previous experience of eating insects. Prior to the experiment, all respondents had some experience of entomophagy. In most cases, however, this involved observing insect-eating behaviors in other people (on television, the internet) rather than through direct personal experience of ingesting insects. Insects are perceived as exotic and are associated with Asian (particularly Vietnamese, Chinese, Cambodian, Indian) or African food, as shown on travel or survival TV shows. The few persons who had themselves tried dishes containing insects before participating in the experiment, tried insects bought by their friends as “souvenirs” from far-away countries.

*“For sure, only not in our culture. In Asia, if I’m not mistaken, Thailand, I think. Chinese markets are what I always associate [with insects], like on travel shows, with tonnes of colourful larvae. It is certainly controversial for Europeans—it would most likely be for me, but [I would be willing to try insects] out of curiosity what that would be like (…).”*

Barriers to eating insects. The first reaction to ingesting insects was disgust. In the participants’ opinion, insects are slimy and evoke associations with filth, basements, and waste. These associations are further reinforced by the image of insects on TV shows, especially on children’s programs. 

Because we associate bugs with something disgusting, bugs in food are more often associated with throwing away food and not eating it. Bugs are eaten by wild animals and not by humans.

*I would be afraid that I would feel the structure of this thing and that it would simply be disgusting: limbs or feelers or something like that. I think everyone is afraid that an insect can come alive in your mouth. Out of some internal fear—they are so disgusting and unpleasant.*

The respondents claimed that in our culture “one does not eat insects—it is as simple as that”. Some participants, even though they were unable to specifically identify what makes insects so disgusting, pointed to the cultural aspects and the importance of upbringing: “we are simply not used to [eating insects]”.

*It is a cultural thing. For us, [insects] are exotic, disgusting, because we have learned to think [about them] this way. [They are] food, as any other type of food, specific for particular regions. And this works this way for us too—we eat pickled cucumbers and sauerkraut, which for some people is simply rotten food. So if we looked at it completely objectively, it seems that eating rotten food is a bigger problem than eating processed insects.*

On the other hand, the participants stated it would be sufficient to “get over oneself and try”. They claimed to see a potential advantage in the wider availability of insects and in their potential to become something commonplace and therefore not rejected by the society in general.

*It is all in your head—we have always been told that an insect is just an insect, it is disgusting, it is a pest. I think this has a huge impact. Insects are not soft and fluffy, but they have hard shells or something, so they don’t look too good either, to be perfectly honest.*

Perceived advantages of eating insects. Many respondents pointed to insects’ high nutritional value as an advantage; they evoked their high protein content and occasionally mentioned other unspecified vitamins and nutrients.

Some respondents mentioned the economic aspects of industrial insect farming. Insects are thought to be inexpensive and easy to produce and transport. Occasionally, the participants described insects as a potential future substitute for meat. Such statements, however, mostly came from vegetarians, who also expressed concerns over the ethical aspects of insect farming. Their answers indicated that they were not certain whether insects were animals. A criterion commonly used for assessing the morality of eating insects was their ability to feel pain. The respondents were not sure whether insects feel pain.

*Perhaps also because, for example, when we kill and eat mammals, they certainly feel more pain than insects—it is as simple as that. Maybe it would be more… humane… this may be the wrong word here… but maybe we could follow that path to reduce the number of animals bred for meat.*

Experiences from the tasting session. The main reason for which the participants decided to try the cookies with insects was curiosity. After seeing a “normal” looking cookie, they were curious whether it tasted different from what they expected from its appearance.

*No, I was wondering if I was going to get anything from the new technologies and if it was going to look strange and resemble God knows what, but I was positively surprised, because it looked tasty. Yes, at first, yes, to some degree, in general, when I saw the labels I thought ‘What did they give me? There is no way I’m trying it.’ But when I had a look, all looked good and this encouraged me to try it. If I had got an insect on my plate, I would never have tried it. When I tried the first one, I completely switched off thinking that I was eating insects.*

The decision to try the products was also made easier by the fact that the products looked appetizing and nice. Another safeguard encouraging the participants to try the cookies was the scientific setting; participating in a research experiment guaranteed the safety and hygiene of the products ingested (“you would not give me anything poisonous to eat”). According to the study participants, one of the potential barriers could be the lack of hygiene linked to eating insects, resulting from the aforementioned associations with filth.

The study participants were unable to precisely identify their expectations and assumptions with regard to the taste of the cookies. They expected the cookies to taste strange or different, “like a bug”, but they were unable to say what they specifically meant. After trying the products, they referred to their own preferences for desserts rather than to the insect content. Their experience was not particularly positive or negative.

Potential for adding insects to everyday diet. The study participants imagined a potential insect consumer to be a young person who is open to new experiences, with a positive attitude, who eats meat but wants to reduce the amount of meat in their diet or to stop eating it completely. In the participants’ opinion, insects could become a fashionable “curiosity” in certain social circles.

The respondents suggested that insect-based food may not be a good idea for the elderly or for vegetarians or vegans (due to the unclear status of insects as animals being able to feel pain). Despite the perceived advantages of insects, their market potential and the positive experiences from the tasting session, most participants claimed that insects were not an appropriate food for them. Yet, some answers suggested a willingness to try insects if they were commercially available. The participants thought, however, that it would rather be a one-off experience than a decision to include insects in their diet on a regular basis. They expressed a wish to reduce the amount of meat and not a need to include other types of meat in their diet. They did not see any direct value for themselves resulting from including insects in their everyday diet.

*It can always be a new taste. I’m curious, I must admit. This can always be a new food form. I doubt that I would be eating [insects] in any large quantities—I’m more interested to just try [them]. I doubt I would try the same form. I have eaten a cookie, maybe [I could eat] an entire cricket, but it only happened once, and that’s probably enough. Yes, I would most likely not include [insects] in my diet, but I would only try [them], in small amounts, out of curiosity.*

The participants believed that the way of serving and the manner of presenting insects could help encourage more people to eat insect-based products. It would be best if insect bodies or parts were not discernible, i.e., if they were added in powder form as a ground protein additive. Some insect types evoke more disgust than others, and the participants stated explicitly that their names should not be provided on product labels or should be indicated in another way that does not elicit negative associations (e.g., larvae). Overcoming the barrier involving associations with filth could be facilitated, in the view of some participants, by the mere presence of products containing insects in grocery shops. They believe that making a product widely available on the market makes people perceive it as being tested and suitable for human consumption. Other respondents pointed to the need to create appropriate insect production safety certificates.

## 4. Discussion

The analysis of the data collected during the experiment revealed a significant effect of Label on product evaluation. Products labelled as containing insects were ingested in smaller quantities and less frequently, regardless of their appearance. In addition, the latency to pick up and eat products was higher in the case of products labelled as containing insects. The effect of label was also found in a study by Mancini and colleagues [14]. The addition of elements imitating insect parts had no effect on consumption levels. This shows that labelling a product as containing insects is, in itself, sufficient to elicit a reluctance to ingest it and results in a decrease in the amount of food ingested. This effect seems to occur regardless of the form in which the insects are served (insect parts or insect flour). Moreover, the type of insect (larvae, crickets, or cockroaches) specified on the label did not affect the quantity of food ingested, which was comparable for all three products. Additionally, when filling in the food evaluation questionnaires, the respondents stated that they were less willing to try products containing insects regardless of insect type and appearance.

While the effect of Label observed in our study is in line with our expectations and confirms the commonly expressed reluctance and aversion to ingesting insect-based products or products with insect content [22,23,24], the fact that no effect of appearance was observed raises several questions. It cannot be ruled out that the appearance of the elements imitating insect parts affected the outcome of the evaluation. The elements were sufficiently small that they did not resemble whole insects or discernible insect body parts (limbs, wings, etc.) It seems that the form of the added elements did not elicit associations with contamination or pollution [44]. At the same time, this effect may confirm earlier observations that adding processed insects elicits fewer negative impressions than adding whole insects (e.g., [20,39]). It may be suggested that the required level of processing need not reduce insects to flour—it is sufficient that consumers are unable to discern insect body parts in the product.

It is interesting, however, that although the respondents were less willing to try products containing insects, there was no difference in scores awarded for sensory qualities. All the products received similar scores on the taste, smell, and appearance scales. Moreover, there was no difference between the groups with regard to the respondents’ willingness to eat those products again. Possible explanations for this may be found in the interviews carried out after the tasting session. The participants stated that they had expected a specific insect taste, and when it turned out that the products labelled as containing insects did not have a new or an unfamiliar taste, they scored the products in the same way as they would score any other cake or pastry. These results are in line with the findings of Sogari and colleagues [43], who showed that both unprocessed and processed insect-based products generate more positive perceptions after tasting compared to expectations. This may suggest that the absence of an unfamiliar taste decreased the novelty effect, thereby reducing neophobia (cf. [27,56]). This is borne out by the fact that in most of the conducted analyses there was no effect of food neophobia as a covariate (cf. [20,57]). The forms of the products (cookie, muffin) were familiar and their taste did not differ from the taste of regular cakes, which may explain why this factor was not observed in the analysis. The only less-commonly known product was the date ball. In this case, food neophobia was only manifested in the amount of food eaten. Participants scoring higher on the food neophobia scale ingested less of the product labelled as containing insects than persons with lower food neophobia levels. Of the three products used in our study, the date ball is the least common and the least widely available in shops. This reduced availability may have elicited a neophobic reaction to an unfamiliar product in some participants [27,56].

The reluctance to try products labelled as containing insects, measured by the latency to pick up food, revealed an effect of the General Neophobia and Variety Seeking Tendency covariates. Participants scoring high on the GN scale picked up the products labelled as containing insects after a longer time than those with low GN levels. Conversely, respondents with high VST scores picked them up sooner than those scoring low on the VST scale. This suggests that the effect of novelty of the products labelled as containing insects manifested itself immediately when participants came into contact with the products. After the product was assessed as safe, however, this effect decreased. The absence of the novelty effect was also observed in the investigative behavior measurements. There were no differences between the groups with regard to the amount of time spent sniffing and looking at the products.

The frequency of water drinking is difficult to explain. There was an interactive effect of Label and Appearance for this variable. When ingesting products in the case of which no insect content was either explicitly indicated on the label or implied by the additional particles, the participants drank water more frequently than when eating products with explicitly stated or implied insect content. It seems that those respondents who ingested non-insect products ate more of the food and therefore needed to drink more water when ingesting it.

The initial reaction to insect-based food that people commonly express verbally is disgust, as confirmed by previous studies (cf. [20,57,58]) and by the interviews conducted in our study. However, this variable was not observed in the behavioral assessment measures, which may be linked, as mentioned above, to the appearance of the elements added to the products that did not elicit associations with contamination or pollution (cf. [44]) and did not resemble whole animal bodies. Low disgust levels may also stem from the experimental setting; in the interviews, the respondents said that they were convinced that in a scientific experiment they would be given safe and hygienic products. They also mentioned that the products were aesthetically pleasing and their dessert form encouraged the participants to try them. This may indicate that serving insects as ingredients in favorite desserts may be a good strategy (cf. [36]), but it may also depend on the food preferences typical of a specific culture. Moreover, it seems that the feeling of disgust reported when thinking about ingesting insects is replaced by other emotions during contact with an aesthetically pleasing product prepared and served in a safe setting.

This may suggest that it is possible to create such products and eating conditions that could help reduce the effect of disgust. If a product is familiar (cf. [14]), has an aesthetically pleasing form, and if consumers are convinced it is safe, such a product may be accepted despite information on insect content (cf. [58,59]). Outside research settings, product safety is determined on the basis of where it is sold. The respondents stated that a product’s widespread commercial availability in shops would encourage them to think that it is safe and that its sales are monitored (cf. [60]). Official safety certificates awarded by appropriate institutions or organizations could have a similar effect. Widespread availability would also convey the impression of a product being widely consumed by others (social proof—[61]). Limited availability, coupled with the fact that they are sold in tourism and pet shops, leads people to still perceive insect-based products to be exotic and foreign.

To conclude, the results of our study confirm the first hypothesis. However, the second hypothesis was not confirmed. The participants, regardless of the appearance of the products, ingested products labelled as containing insects in smaller amounts. In the interviews, the respondents implied that the information about insect content was in itself enough to evoke reluctance and doubts as to whether they should try the product. They pointed out that images of insects on product packaging would also create a negative impression and suggested that certain types of insects (e.g., mealworm larvae) were more disgusting than others.

It seems, however, that it is without consequence whether insects are added in the form of small pieces or flour, as long as whole insects or their body parts are not easily discernible. It is a good strategy to add insects to well-liked products. Insects should be added to familiar products, with familiar taste/smell/texture, and the elements added should not change those properties. The products themselves should have an aesthetically pleasing appearance. All those measures may help convince consumers that such products are safe. In this way, the consumers’ preferences for specific products may be generalized to insect-based products.

The study has many implications for management and marketing strategies. First, the results of the study showed that the manner of communicating information on insect-based ingredients has a huge impact on the perception of the product and its future marketing success. The presence of such information is enough to reduce interest in the product. Therefore, placing such products on the market should be preceded by extensive consumer research conducted with a view to selecting the right message and labelling to eliminate that negative effect. Second, to ensure a product’s marketing success, it is important to select the right target group. Our research has shown that the group most open to insect-based food are experimenters and variety seekers; on the other hand, the group most reluctant to accept such products are people with high levels of neophobia. This result suggests that an effective positioning strategy of a product containing insect ingredients should refer to “variety seeking” or “experimenting”. This study also showed no differences in the evaluation of taste regardless of whether the products had been labelled as containing insects or not. At the same time, unwillingness to try products labelled as containing insects suggests that the problem does not lie in the taste of the product (which has also been confirmed by other studies), but rather in some sort of prejudice against products that contain insects. This is linked to the third implication: it is probably worthwhile to place such products on the market using a “sampling strategy”, such as food tasting campaigns in shops, which can help consumers overcome their mental block and try these products.

A very significant aspect of our study is the fact that we used several behavioral measures and not only participants’ declarations, as is the case with many studies conducted to date. In doing so, we were able to observe real-life multidimensional reactions to the products used. The interviews conducted after the behavioral assessment phase proved helpful for interpreting the quantitative results and provided ideas for future research and practical solutions.

The study, however, has certain limitations. The study population was characterized by a significant gender imbalance, which prevented us from analyzing the effect of the gender variable. Nevertheless, there was no theoretical basis for assuming such an effect, and we strove to ensure that the gender ratio was identical in each group. The same applied to the remaining demographic data, i.e., level of education and age, as well as dietary preferences—no differences were observed for those variables between the groups. Another limitation was that the majority of participants were students, which may reduce the ability to generalize the results to the general population. However, the participants in our study were full-time as well as evening-course students. The latter represent a broader spectrum of population, as they are often older than full-time students; they often work full-time and have families, which may reduce the effect of the specificity of the study group. Nevertheless, future research should be conducted on a more socio-economically diverse sample to help identify the general mechanisms underlying the phenomenon examined and to ensure the relevance of the findings for marketing strategies. Another important factor that should be considered is personal food preference, especially attitudes towards meat consumption. The results of the interviews show that people following a vegetarian diet are not sure about the appropriateness of consuming insects, as they do not fully understand whether arthropods are able to feel pain, etc.

It is crucial to examine cultural differences between the populations (e.g., participants from different countries), as populations may differ in their food preferences. It is possible that this factor substantially influences acceptance of insects as food and the choice of specific products. Cross-cultural comparative studies (including Asian countries) would also shed light on the universality of the mechanisms underlying the acceptance/rejection of entomophagy.

With regard to the possibility of applying research results in practice, future studies should examine other measures identifying different personal characteristics, such as the tendency to take risks, curiosity, and the need for exploration, as well as variables related to health and moral values.

## Figures and Tables

**Table 1:** Experimental group set-up. The products in groups 1A and 2A (no visual clues suggestive of insect content) and in groups 1B and 2B (presence of visual clues suggestive of insect content) looked identical, but differed in the information provided on the labels.

		**Label**—Explicit Information about the Presence or Absence of Insect Content	
		**No Insect Content** **“1”**	**Insect Content** **“2”**
**Appearance**—presence or absence of visual features of the product suggestive of insect content	No visual clues suggestive of insect content “A”	Condition 1A	Condition 2A
	Visual clues suggestive of insect content “B”	Condition 1B	Condition 2B

**Table 2:** Characteristics of the study groups. Abbreviation sd refers to the standard deviation.

Variable	Group 1A *N* = 24	Group 1B *N* = 25	Group 2A *N* = 25	Group 2B *N* = 25	Statistics
Sex (women—F; men—M)	18F/6M	22F/3M	19F/6M	21F/4M	χ^2^(3) = 1.893, *p* = 0.959
Age—mean (sd)	22.1 (3.7)	22.5 (5.3)	22.2 (5.4)	23 (6.9)	F(3; 95) = 0.139, *p* = 0.936
Education (secondary/student/higher)	0/21/3	2/19/4	1/20/4	1/22/2	χ^2^(5) = 3.071, *p* = 0.800
Diets (meat/non-meat)	16/8	13/12	11/14	14/11	χ^2^(9) = 10.637, *p* = 0.301
Food Neophobia—mean (sd)	32.3 (8.5)	32.2 (11.4)	30.8 (11.8)	29.2 (12.3)	F(3; 95) = 0.435, *p* = 0.729
General Neophobia—mean (sd)	26.0 (10.8)	30.4 (11.0)	32.0 (11.5)	27.6 (10.8)	F(3; 95) = 1.499, *p* = 0.220
Food Technology Neophobia—mean (sd)	46.6 (12.7)	46.4 (10.1)	51.3 (13.9)	50.4 (13.5)	F(3; 95) = 0.980, *p* = 0.405
Variety Seeking Tendency—mean (sd)	30.8 (5.6)	31.0 (6.4)	31.7 (6.0)	31.2 (7.3)	F(3; 95) = 0.094, *p* = 0.963
Disgust—mean (sd)	15.2 (5.6)	16.1 (4.4)	14.1 (4.6)	15.7 (4.8)	F(3; 95) = 0.777, *p* = 0.510

## Appendix A

**Descriptive Statistics of All Quantitative Measures Registered in This Study.**				
	**Group**	**N**	**Mean**	**Standard Deviation**
Latency to pick up food sample	1A	24	10.743	7.751
	1B	25	20.191	18.22
	2A	25	35.315	43.451
	2B	25	21.604	18.359
Latency to begin eating	1A	24	22.018	15.059
	1B	25	28.31	17.194
	2A	25	33.991	18.752
	2B	25	34.015	19.735
Frequency of sniffing food samples	1A	24	3.708	2.136
	1B	25	4.32	1.52
	2A	25	3.333	2.099
	2B	25	4.08	2.1
Frequency of looking at food samples	1A	24	12	6.386
	1B	25	12.76	9.791
	2A	25	8.375	5.046
	2B	25	11.28	8.503
Time spent sniffing food samples	1A	24	6.21	4.728
	1B	25	5.992	3.505
	2A	25	5.601	5.158
	2B	25	4.596	2.404
Time spent looking at food samples	1A	24	36.309	25.171
	1B	25	44.262	36.747
	2A	25	28.044	20.075
	2B	25	34.713	25.275
Amount of eaten sample A	1A	24	43.681	35.925
	1B	25	39.061	39.095
	2A	25	14.543	19.209
	2B	25	16.305	21.256
Amount of eaten sample B	1A	24	45.169	40.854
	1B	25	39.27	35.224
	2A	25	26.29	30.39
	2B	25	27.511	30.362
Amount of eaten sample C	1A	24	80.306	32.5
	1B	25	77.298	37.458
	2A	25	50.038	44.672
	2B	25	37.981	38.167
Time spent eating	1A	24	402.23	153.93
	1B	25	405.18	196.216
	2A	25	316.694	139.009
	2B	25	327.623	131.485
Frequency of the eating bouts	1A	24	9	4.181
	1B	25	8.72	6.724
	2A	25	5.875	4.875
	2B	25	5.84	4.22
Frequency of drinking water	1A	24	36.309	25.171
	1B	25	44.262	36.747
	2A	25	28.044	20.075
	2B	25	34.713	25.275
